# Effect of Diets Supplemented with Different Sources of Astaxanthin on the Gonad of the Sea Urchin *Anthocidaris crassispina*

**DOI:** 10.3390/nu4080922

**Published:** 2012-08-13

**Authors:** Juan Peng, Jian-Ping Yuan, Jiang-Hai Wang

**Affiliations:** Guangdong Provincial Key Laboratory of Marine Resources and Coastal Engineering, School of Marine Sciences, Sun Yat-Sen University, Guangzhou 510275, China; Email: yuanjp@mail.sysu.edu.cn (J.-P.Y.); wangjhai@mail.sysu.edu.cn (J.-H.W.)

**Keywords:** astaxanthin, *Haematococcus pluvialis*, *Chorella zofingiensis*, sea urchin, gonad, *Anthocidaris crassispina*

## Abstract

The effect of the microalgae *Haematococcus pluvialis* and *Chorella zofingiensis*, and synthetic astaxanthin on the gonad of the sea urchin* Anthocidaris crassispina* was studied. The basal diet was supplemented with *H. pluvialis*, *C. zofingiensis*, or synthetic astaxanthin, at two levels of astaxanthin (approximately 400 mg/kg and 100 mg/kg), to obtain the experimental diets HP1, HP2, CZ1, CZ2, AST1, and AST2, respectively, for two months of feeding experiment. The results showed that the concentrations of astaxanthin in the gonads of the sea urchins fed these experimental diets ranged from 0.15 to 3.01 mg/kg dry gonad weight. The higher astaxanthin levels (>2.90 mg/kg) were found in the gonads of the sea urchins fed the diets HP1 (containing 380 mg/kg of astaxanthins, mostly mono- and diesters) and AST1 (containing 385 mg/kg of synthetic astaxanthin). The lowest astaxanthin level (0.15 mg/kg) was detected in the gonads of the sea urchins fed the diet CZ2 (containing 98 mg/kg of astaxanthins, mostly diesters). Furthermore, the highest canthaxanthin level (7.48 mg/kg) was found in the gonads of the sea urchins fed the diet CZ1 (containing 387 mg/kg of astaxanthins and 142 mg/kg of canthaxanthin), suggesting that astaxanthins, especially astaxanthin esters, might not be assimilated as easily as canthaxanthin by the sea urchins. Our results show that sea urchins fed diets containing astaxanthin pigments show higher incorporation of these known antioxidant constituents, with the resultant seafood products therefore being of potential higher nutritive value.

## 1. Introduction

The sea urchin* Anthocidaris crassispina* is commonly found in the coastal waters of southern China and is an economically important species in China [1,2,3]. The gonads of the sea urchins are a highly valued seafood and are considered a prized delicacy in many countries, even as a staple food in Chile and Japan [2,4,5]. The quality of gonad depends on its mass and appearance, especially its color, which is one of the major factors affecting the marketability of sea urchins [6], and is determined by the composition of carotenoids accumulated in the gonads of sea urchins [7]. 

The red ketocarotenoid astaxanthin, 3,3′-dihydroxy-β,β-carotene-4,4′-dione, is one of the major carotenoids naturally found in sea urchins and has been added to feed to improve gonad color of the sea urchins [8,9]. In addition, astaxanthin, with two carbonyl groups, two hydroxyl groups, and eleven conjugated ethylenic double bonds, may act as a strong antioxidant by donating electrons, reacting with free radicals, and terminating free radical chain reaction in a wide variety of living organisms, and thus has an antioxidant activity as high as ten times more than other carotenoids, such as zeaxanthin, lutein, canthaxanthin, and β-carotene, and over 100 times more than α-tocopherol [10]. Astaxanthin has been reported to have potential health-promoting effects in the prevention and treatment of various diseases, such as cancers, chronic inflammatory diseases, metabolic syndrome, diabetes, diabetic nephropathy, cardiovascular diseases, gastrointestinal diseases, liver diseases, neurodegenerative diseases, eye diseases, skin diseases, and exercise-induced fatigue [10]. Therefore, the gonads of sea urchins containing astaxanthin have a higher nutritive value. Besides being chemically synthesized, astaxanthin can be produced by the green microalgae *Haematococcus pluvialis* and *Chorella zofingiensis* [8]. *H. pluvialis* is the richest source of natural astaxanthin among all algal species reported [11,12,13], and *C. zofingiensis* is proposed as a promising producer of astaxanthin because of its capability of accumulating astaxanthin under heterotrophic conditions [14,15,16]. 

The use of algal biomass as a coloring agent has been investigated [17,18,19,20]. Shpigel *et al*. [18] achieved satisfactory color in gonads of the sea urchin *Paracentrotus lividus* by supplementing the prepared diet with the macroalga *Ulva lactuca*. Chatzifotis *et al*. [19] found that *H. pluvialis* in diets for red porgy *Pagrus pagrus* had a significant effect on skin pigmentation. The use of synthetic carotenoids is much more common because they are easy to get [21]. However, there are few studies on the effects of natural astaxanthin as well as synthetic astaxanthin on the gonads of sea urchins. Therefore, this study investigated the effects of different sources of astaxanthin on the gonads of the sea urchin *A. crassispina* using compound feeds containing the microalgae *H. pluvialis* or *C. zofingiensis* as natural astaxanthin sources, and synthetic astaxanthin. In this study, the concentrations of carotenoids in the gonads were determined after feeding the experimental diet to sea urchins. 

## 2. Materials and Methods

### 2.1. Collection and Maintenance

Sea urchin *A. crassispina* was collected by scuba diving to a depth of 4 to 5 m in Port Shelter, Hong Kong in May. The macroalga *Sargassum hemiphyllum*, the natural food of the sea urchin, was also collected. After collection, the sea urchins were maintained in 200-L tanks in the laboratory at 25 °C and 32‰ salinity in an aerated, closed-circuit filtration system without feeding for 1 week to ensure that all would be of similar nutritional status [22,23]. Eighty sea urchins with a mean diameter of 37.8 ± 0.2 mm were randomly divided into eight groups, including an Initial group and seven feeding experimental groups, the initial diameters were not significantly different among these groups (One Way ANOVA, *F* = 0.034, *p* = 0.999). The sea urchins in the Initial group were sacrificed and analyzed at the beginning of the feeding experiment. In seven experimental groups, each sea urchin was housed in an individual plastic container (15 × 12 × 9 cm^3^) placed into a 115-L experimental tank (10 containers per tank) containing filtered seawater at 25 °C. Each container was supplied independently with seawater at 0.1 L/min. A 12/12 h light/dark photoperiod and 20 µmol photons m^−2^ s^−1^ light intensity measured at the surface of the tanks were used. The laboratory feeding experiments were conducted for two months following the initial week of starvation (*n* = 10 per diet). Freshly prepared diet was added, uneaten diet and feces were removed every 2 days, and seawater was changed weekly [23].

### 2.2. Experimental Diets

#### 2.2.1. Astaxanthin Sources

Synthetic astaxanthin (purity of 98%) was obtained from Sigma-Aldrich, St. Louis, MO, USA. For natural astaxanthin sources, the microalgae *H. pluvialis* and *C. zofingiensis* were cultured as described by Peng *et al*. [24] and Wang and Peng [16], respectively. The algal cells were harvested by centrifuging the culture samples at 3500× *g* at 4 °C for 5 min and the supernatant was discarded. The algal cells were rinsed with distilled water twice and dried in a DW3 freeze-drier (Heto Dry Winner, Denmark). The dried algal cells were stored at −20 °C for subsequent carotenoid analysis and diet preparation.

#### 2.2.2. Diet Preparation

The basal diet without adding astaxanthin from any source (Basal diet) was used as a control group. The basal diet was composed of the macroalga *S. hemiphyllum*, which has been used to feed sea urchins [25], fish meal, flour, and agar. Table 1 is a list of the percentage of raw ingredients in seven experimental diets. 

*S. hemiphyllum* was rinsed with distilled water twice, freeze-dried, and ground into powder in a Wiley Mill. Fish meal, flour, and agar were purchased from a local market. Six experimental groups (HP1, HP2, CZ1, CZ2, AST1, and AST2) were fed the basal diet, supplemented with three different astaxanthin sources (the microalgae *H. pluvialis* and *C. zofingiensis*, and synthetic astaxanthin) at two levels of astaxanthins (approximately 400 and 100 mg/kg [9]), respectively. The levels of astaxanthins in the microalgae were first determined by HPLC (see section 2.3.2) and the diet composition was formulated accordingly (Table 1). The diets were freshly prepared every 7 days, according to Shpigel, M. *et al*. [26]. Briefly, 25 g of agar was dissolved in about 300 mL of distilled water, then adding 5 g of fish meal, 20 g of *S. hemiphyllum* powder, and 50 g of flour supplemented with different sources of astaxanthin, and mixing and kneading thoroughly. The dough was formed into about 100 diet flakes using a circular mold, and then dried at 50 °C for 24 h. Each dried diet flake (about 1 g) was kept in an opaque, sealed plastic bag and stored at −20 °C for subsequent feeding. Three dried diet flake samples were taken for the analysis of carotenoids within 12 h. The sea urchins were fed daily on an *ad libitum* basis for two months. 

**Table 1:** Experimental diets.

Compositions (%)	Basal Diet	HP1	HP2	CZ1	CZ2	AST1	AST2
Dried fish meal	5.0	5.0	5.0	5.0	5.0	5.0	5.0
Dried agar	25.0	25.0	25.0	25.0	25.0	25.0	25.0
Dried *S. hemiphyllum*	20.0	20.0	20.0	20.0	20.0	20.0	20.0
Dried Flour	50.0	47.5	49.4	19.7	42.4	50.0	50.0
Dried *H. pluvialis* ^1^	–	2.5	0.6	–	–	–	–
Dried *C. zofingiensis* ^2^	–	–	–	30.3	7.6	–	–
Synthetic astaxanthin	–	–	–	–	–	0.04	0.01

^1^ Total concentration of astaxanthins (including astaxanthin, astaxanthin monoesters, and astaxanthin diesters) in *H. pluvialis* determined by HPLC (see section 2.3.2) was 16.19 g/kg; ^2^ Total concentrations of astaxanthins in *C. zofingiensis* determined by HPLC were 1.32 g/kg.

### 2.3. Analysis

#### 2.3.1. Determination of Gonad Index

The diameter and weight of the sea urchins from the Initial group and the experimental groups were measured. These sea urchins were starved for 2 days prior to measuring weight. After dissection, the gonads of the sea urchins were placed on laboratory tissue to remove excess seawater, weighed, and then were dried in the DW3 freeze-drier for 48 h. The gonad index of the sea urchins was calculated as the percentage of gonad wet weight divided by the drained wet weight of the intact sea urchin [23,27].

#### 2.3.2. Determination of Carotenoid Concentrations

The dried gonads and dried diet flakes were ground into powder with a porcelain mortar and pestle. Carotenoids in samples (microalgae, diet flake powder, and gonad powder) were extracted with acetone according to the previous methods [12,24,28]. Briefly, known weights of dried samples (10 mg for diet flakes and microalgae, 100 mg for gonads) placed in a mortar were ground with a pestle and 5 mL of acetone as extraction solvent was added under liquid nitrogen. The mixture of sample and extraction solvent was then separated by centrifugation at 12,000 *g* at 4 °C for 5 min, and the supernatant was collected. The extraction procedure was repeated until the sample debris was almost colorless [3], and the extractions were pooled. The whole process was carried out in darkness. 

Aliquots of pigment extracts (20 µL) were separated by a Waters HPLC system (Waters, Milford, CT, USA) equipped with a 1525 binary pump and a 2996 photodiode array detector, using a Waters Spherisorb^®^ ODS2 reverse-phase C18 column (4.6 mm × 250 mm, 5 μm) according to the modified method described by Wang and Peng [16]. All injections were repeated 3 times. A gradient mobile phase consisting of solvent A (acetonitrile/methanol/0.1 M Tris-HCl, pH 8.0, 84:2:14, v:v) and solvent B (methanol/ethyl acetate, 68:32, v:v) was used: 0–15 min, 0–100% B; 15–25 min, 100% B; 25–28 min, 100%–0% B; The flow rate was 1.2 mL/min. Chromatographic peaks were measured at a wavelength of 450 nm. The concentrations of astaxanthin, canthaxanthin, β-carotene, lutein, fucoxanthin, and echinenone were determined using the external standard method. Because no isocryptoxanthin and astaxanthin ester standards were available in the present work, isocryptoxanthin and astaxanthin esters were measured by comparing the peak area with the β-carotene and astaxanthin standards, respectively [24]. Astaxanthin, canthaxanthin, β-carotene, and lutein were purchased from Sigma (Milwaukee, WI, USA). Fucoxanthin and echinenone were obtained from ChromaDex (Irvine, CA, USA). HPLC-grade acetonitrile, ethyl acetate, and methanol were obtained from BDH (Poole, UK). 

### 2.4. Statistical Analysis

All data were expressed as mean ± standard error (SE). The significance of differences among mean values was determined using analysis of variance (one-way ANOVA), followed by Tukey’s multiple range test. A significance level of *p* < 0.05 was considered significant.

## 3. Results

### 3.1. Carotenoids in Experimental Diets

**Figure 1:**
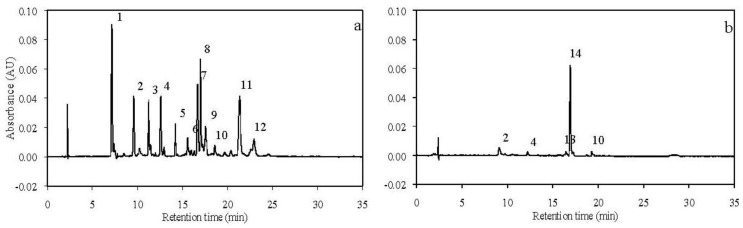
Representative HPLC chromatograms from a diet (**a**) and the gonad (**b**) of the sea urchins fed the diet. Peaks identified were fucoxanthin (1); astaxanthin (2); lutein (3); canthaxanthin (4); chlorophyll *b* (5); chlorophyll *a* (6); astaxanthin monoesters (7–9); β-carotene (10); astaxanthin diesters (11,12); isocryptoxanthin (13); and echinenone (14).

The concentrations of carotenoids, including free astaxanthin, astaxanthin monoesters, astaxanthin diesters, lutein, canthaxanthin, echinenone, isocryptoxanthin, fucoxanthin, and β-carotene in the seven experimental diets were determined by HPLC (Figure 1a), and the results are shown in Table 2. 

**Table 2:** Concentrations of carotenoids in experimental diets (mg/kg).

Carotenoids	Basal Diet	HP1	HP2	CZ1	CZ2	AST1	AST2
Astaxanthins	–	380.59	95.92	387.27	97.87	385.04	92.13
free	ND	10.47 ± 0.07	2.42 ± 0.03	8.77 ± 1.00	2.25 ± 0.00	385.04 ± 14.60	92.13 ± 4.19
monoesters	ND	183.16 ± 10.92	46.27 ± 1.52	62.24 ± 2.40	15.72 ± 1.01	ND	ND
diesters	ND	186.96 ± 14.42	47.23 ± 1.83	316.26 ± 3.55	79.90 ± 3.70	ND	ND
Lutein	0.56 ± 0.11	18.15 ± 3.07	4.71 ± 0.13	40.23 ± 2.95	6.80 ± 1.03	ND	ND
Fucoxanthin	86.34 ± 9.85	80.74 ± 4.47	83.90 ± 3.25	80.53 ± 1.12	84.77 ± 0.92	85.53 ± 1.43	84.34 ± 2.48
Canthaxanthin	ND	10.88 ± 1.38	2.87 ± 0.19	142.23 ± 3.90	21.82 ± 2.50	ND	ND
β-Carotene	8.93 ± 0.09	15.60 ± 0.01	8.10 ± 0.05	14.22 ± 0.19	8.20 ± 0.00	8.67 ± 0.08	8.34 ± 0.00
Isocryptoxanthin	ND	ND	ND	ND	ND	ND	ND
Echinenone	ND	ND	ND	ND	ND	ND	ND

ND: not detected.

The result showed that the concentrations of astaxanthin and astaxanthin esters varied in different experimental diets in accordance with the sources and levels of astaxanthin. The diets HP1, HP2, CZ1, and CZ2 contained remarkably high amounts of astaxanthin esters, while only a small quantity of free astaxanthin was found in these diets. The results also showed that the microalgae *H. pluvialis* and *C. zofingiensis* contained 16.19 and 1.32 g/kg of total astaxanthins, respectively, including free astaxanthin, astaxanthin monoesters and diesters. In addition, the diets HP1, HP2, CZ1, and CZ2 also contained lutein, canthaxanthin, and β-carotene, especially the diet CZ1, which contained the highest amount of canthaxanthin (142.23 mg/kg) from *C. zofingiensis*, and no isocryptoxanthin, and echinenone were detected in these diets. Furthermore, fucoxanthin from the macroalga *S. hemiphyllum* was also detected in seven experimental diets (Table 2).

### 3.2. Carotenoids in the Gonads of the Sea Urchin *A. crassispina*

The concentrations of carotenoids, including astaxanthin, canthaxanthin, isocryptoxanthin, echinenone, and β-carotene in the gonads of initial sea urchins and the sea urchins fed the seven experimental diets were determined by HPLC (Figure 1b), and the results are shown in Table 3. The results indicated that astaxanthin was present in the gonads of the sea urchins fed the diets with added astaxanthin, and no astaxanthin was detected in the gonads of initial sea urchins and the sea urchins fed the basal diet, indicating that astaxanthin accumulating in the gonads came from the diets containing astaxanthin.

The concentrations of astaxanthin in the gonads of the sea urchins fed astaxanthins varied obviously and ranged from 0.15 to 3.01 mg/kg dry gonad (Table 3). The higher astaxanthin concentrations (2.93–3.01 mg/kg) were detected in the gonads of the sea urchins fed the diet HP1, which contains 381 mg/kg of free and esterified astaxanthins, and the diet AST1, which contains 385 mg/kg of synthetic astaxanthin. The lower astaxanthin concentrations (0.15–0.26 mg/kg) were detected in the gonads of the sea urchins fed the diets CZ2 and CZ1, whose astaxanthin levels were similar to that of the diets HP2 and HP1. It was notable that the gonads of sea urchins fed 380.6–387.3 mg/kg of astaxanthins were found to have significantly higher levels (*p* < 0.05) of astaxanthin (1.7–4.8 fold) than that fed 92.1–97.9 mg/kg of astaxanthins from three sources, respectively.

**Table 3:** Concentrations of carotenoids in the gonads of sea urchins (mg/kg).

Carotenoids	Initial	Basal Diet	HP1	HP2	CZ1	CZ2	AST1	AST2
Astaxanthin	ND	ND ^e^	3.01 ± 0.06 ^a^	1.09 ± 0.05 ^b^	0.26 ± 0.01 ^d^	0.15 ± 0.01 ^d^	2.93 ± 0.04 ^a^	0.61 ± 0.01 ^c^
monoester	ND	ND	ND	ND	ND	ND	ND	ND
diester	ND	ND	ND	ND	ND	ND	ND	ND
Lutein	ND	ND	ND	ND	ND	ND	ND	ND
Fucoxanthin	0.21 ± 0.01	ND	ND	ND	ND	ND	ND	ND
Canthaxanthin	1.84 ± 0.05	ND ^d^	1.09 ± 0.49 ^c^	0.46 ± 0.01 ^cd^	7.48 ± 0.35 ^a^	2.79 ± 0.42 ^b^	ND ^d^	ND ^d^
Isocryptoxanthin	1.82 ± 0.04	0.60 ± 0.02 ^c^	0.71 ± 0.01 ^bc^	0.95 ± 0.24 ^ab^	0.48 ± 0.06 ^c^	0.67 ± 0.02 ^bc^	0.51 ± 0.01 ^c^	1.08 ± 0.07 ^a^
Echinenone	45.96 ± 1.31	12.7 ± 1.72 ^b^	23.52 ± 0.12 ^a^	21.77 ± 0.12 ^a^	15.41 ± 2.23 ^a^	14.11 ± 0.24 ^b^	15.34 ± 0.76 ^a^	9.56 ± 1.87 ^b^
β-carotene	3.61 ± 0.28	0.89 ± 0.04 ^de^	1.96 ± 0.01 ^a^	1.69 ± 0.06 ^b^	0.91 ± 0.03 ^d^	0.56 ± 0.05 ^f^	1.27 ± 0.05 ^c^	0.68 ± 0.14 ^ef^

ND: not detected; ^a,b,c,d,e,f^ means with different superscript letters within the same carotenoid groups differ significantly (*p* < 0.05).

The results showed that canthaxanthin, another ketocarotenoid from the algae *H. pluvialis* and *C. zofingiensis*, could also be accumulated in the gonads of sea urchins, and the highest canthaxanthin level (7.48 mg/kg) was found in the gonads of the sea urchins fed the diet CZ1, which contains 142.23 mg/kg of canthaxanthin derived from *C. zofingiensis*. Echinenone, the primary coloration carotenoid accumulating in the gonads of wild sea urchins, was found to still be the dominant carotenoid in the gonads of sea urchins after two months of feeding, although the concentrations of echinenone were much lower (9.56–23.52 mg/kg) than that of the initial sea urchins (45.96 mg/kg).

A similar decrease in the concentrations of isocryptoxanthin and β-carotene was also found. The concentrations of isocryptoxanthin (0.48–1.08 mg/kg) in the gonads of the sea urchins fed different experimental diets, in which no isocryptoxanthin was detected, were markedly lower than that of the initial sea urchins (1.82 mg/kg). Furthermore, the concentrations of β-carotene (0.56–1.96 mg/kg) in the gonads of sea urchins fed different experimental diets, in which different levels of β-carotene (Table 2) were detected, were evidently lower than that of the initial sea urchins (3.61 mg/kg). In addition, no lutein and fucoxanthin were detected in the gonads of sea urchins fed different experimental diets, in which a small quantity of lutein and a large quantity of fucoxanthin were present.

### 3.3. The Gonad Index of Sea Urchins

The diameter, total wet weight, gonad wet weight, and gonad index of sea urchins were determined and the results are shown in Table 4. 

**Table 4:** Gonad index and test diameter of sea urchins.

	Initial	Basal Diet	HP1	HP2	CZ1	CZ2	AST1	AST2
Diameter (mm)	37.8 ± 1.4	38.2 ± 1.2	38.3 ± 1.1	38.8 ± 1.0	38.3 ± 0.7	38.4 ± 0.7	38.3 ± 0.3	38.3 ± 0.6
Total weight (g)	29.9 ± 0.3	30.3 ± 2.8	30.7 ± 2.5	32.3 ± 2.2	31.0 ± 1.7	32.0 ± 1.7	30.2 ± 0.6	30.7 ± 1.4
Gonad weight (g)	1.6 ± 0.6	2.6 ± 0.4	3.5 ± 0.5	3.6 ± 0.5	3.3 ± 0.3	2.8 ± 0.3	3.2 ± 0.3	3.5 ± 0.3
Gonad index (%)	5.2 ± 1.2	8.5 ± 0.8	11.2 ± 0.8	10.9 ± 1.0	10.7 ± 0.7	8.7 ± 0.9	10.5 ± 0.8	11.2 ± 0.9

The results showed that the diameters (One Way ANOVA, *F* = 0.040, *p* = 1.000) and weights (One Way ANOVA, *F* = 0.164, *p* = 0.985) of these sea urchins were not significantly different at the end of the experiment. By contrast, the gonad weight and gonad index increased remarkably for all experiment diet groups (Table 4), and the higher gonad index (10.5%–11.2%) was found in the sea urchins fed the diets supplemented with astaxanthin with the exception of the diet CZ2 (8.7%), in comparison with the sea urchins fed the basal diet (8.5%), but the gonad weight (One Way ANOVA, *F* = 0.994, *p* = 0.437) and the gonad index (One Way ANOVA, *F* = 2.085, *p* = 0.068) were not significantly different in seven experimental diets.

## 4. Discussion

The accumulation of astaxanthin in the gonad of the sea urchin *A. crassispina* varied significantly with the sources and levels of astaxanthin added to the experimental diets. There was a higher astaxanthin accumulation in the gonads of sea urchins fed the diets supplemented with a higher level of astaxanthin. Similar findings were observed by Hávardsson *et al*. [9], who found that the sea urchins fed synthetic astaxanthin showed an increase in the astaxanthin concentration in the gonads, but a smaller quantity (0.05–1.00 mg/kg) of astaxanthin was incorporated in the gonads, even in the sea urchins fed high level of astaxanthin (500 mg/kg). The artificial sources of astaxanthin presents three configurational isomers, two enantiomers (3*S*, 3′*S* and 3*R*, 3′*R*) and a meso form (3*R*, 3′*S*), and the 3*S*, 3′*S* stereoisomer is the main form found in natural sources. Therefore, it was possible that synthetic astaxanthin might not be assimilated as easily as natural sources. Hávardsson *et al*. [9] suggested that synthetic astaxanthin should be ruled out as a feasible ingredient for increasing gonad carotenoids in sea urchins. In the present study, a higher concentration of astaxanthin (2.93 mg/kg) in the gonads was observed in the sea urchins fed the diets containing 385 mg/kg of synthetic astaxanthin. This difference in astaxanthin concentration might be explained with differences in nutritional status [9] and sea urchin species. 

The higher astaxanthin levels (>2.9 mg/kg) were found in the gonads of sea urchins fed the diets HP1 and AST1, and the lower astaxanthin levels (<0.3 mg/kg) were detected in the gonads of the sea urchins fed the diets CZ1 and CZ2. The results indicated that the absorption of astaxanthins by sea urchins from the alga *C. zofingiensis*, which contains 16.1% of astaxanthin monoesters and 81.7% of astaxanthin diesters, was much lower than that from the alga *H. pluvialis*, which contains 48.1% of astaxanthin monoesters and 49.1% of astaxanthin diesters, demonstrating that the accumulation of astaxanthin in the gonads was different when supplied as free, monoesters, or diesters in the diets. A similar study has been reported for salmon fed free astaxanthin and astaxanthin esters [29], and non-esterified astaxanthin was considered to be readily absorbable. No astaxanthin esters were found in the gonads of the sea urchins fed these diets, indicating that astaxanthin esters in these diets might be hydrolyzed before accumulating in the gonads after being absorbed by the sea urchins, or might not be easily assimilated by the sea urchins, especially astaxanthin diesters. Analyzing further the guts and feces would provide information on this.

Moreover, canthaxanthin in diets could be also accumulated in the gonads of sea urchins, and the deposition of canthaxanthin in the gonads of sea urchins was easier than that of astaxanthins, especially astaxanthin esters. The accumulation difference of canthaxanthin and astaxanthins in the gonads of sea urchins might be related to the difference in the absorption, transport, deposition or metabolism of canthaxanthin and astaxanthins [30].

**Figure 2:**
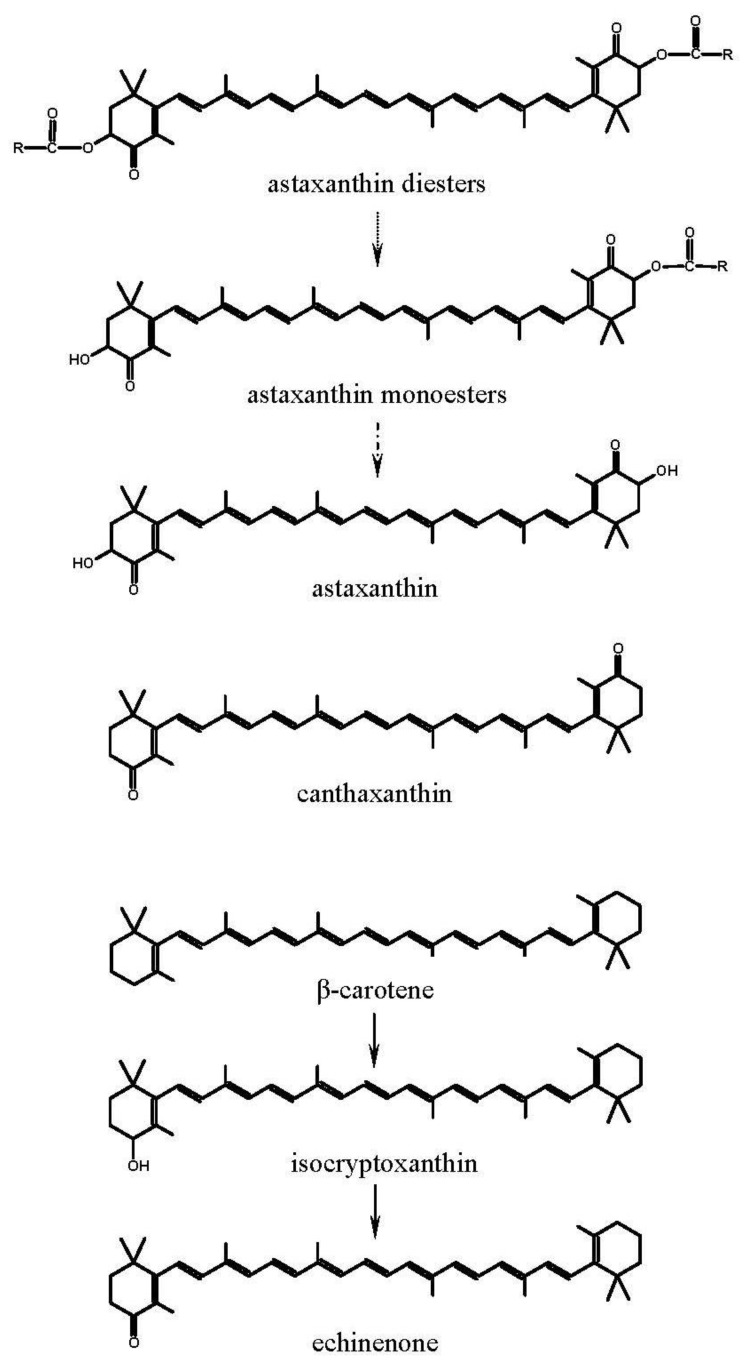
Chemical structures or proposed metabolic pathways of carotenoids in sea urchins.

The concentrations of echinenone in the gonads of the sea urchins fed different experimental diets remarkably reduced in the present study. This is probably due to the lack of a suitable metabolic precursor for production of echinenone [9], and suggests that higher oxidation state carotenoids such as astaxanthin and canthaxanthin cannot be metabolized by these sea urchins to echinenone [26]. It had been suggested that β-carotene was bioconverted to echinenone via isocryptoxanthin in the sea urchins (Figure 2) [7,31]. Astaxanthin could be ruled out as a possible precursor and was not metabolized into echinenone [9,31]. However, the decreases in carotenoid concentrations might not indicate a decrease in the amount of carotenoids in the gonads, because the gonad weight increased for all experiment diet groups (Table 4). The results indicated that the amounts of echinenone in the gonads of the sea urchins fed the diets HP1 and HP2 were larger than that of the Initial sea urchins (data not shown), indicating that the alga *H. pluvialis* in the diets HP1 and HP2 could meliorate the accumulation of echinenone (21.77–23.52 mg/kg) in comparison with the basal diet and the diets CZ1, CZ2, AST1, and AST2 (9.56–15.41 mg/kg), although the alga *H. pluvialis* did not contained echinenone.

Furthermore, the concentrations of isocryptoxanthin and β-carotene in the gonads of the sea urchins fed different experimental diets, in which different levels of β-carotene and no isocryptoxanthin were detected, were markedly lower than that of the initial sea urchins after two months of feeding. The diets containing the alga *C. zofingiensis* (CZ1 and CZ2) could resist the accumulation of β-carotene in comparison with the diets HP1 and HP2. Tang *et al*. [32] found that canthaxanthin had an antagonistic effect on β-carotene uptake in ferrets, demonstrating the interactions between dietary carotenoids in mammals. Liyana-Pathirana *et al*. [5] reported a significant reduction in total carotenoid content in cultured sea urchins after feeding on an artificial diet for 9 weeks. The reduction in these carotenoids may be due to natural breakdown or conversion into other metabolic products [9].

In addition, although fucoxanthin and lutein were present in the experimental diets, no fucoxanthin and lutein were found in the gonads of the sea urchins fed different experimental diets containing fucoxanthin and/or lutein. Suckling *et al*. [33] reported that the addition of lutein and zeaxanthin failed to improve gonad color in their study. Kawakami *et al*. [34] found that fucoxanthin was a major carotenoid in the viscera and could not be detected in the gonad of the sea urchin. These studies indicated that the sea urchins lacked the ability to accumulate fucoxanthin and lutein in the gonad.

The results showed that the gonad index of the sea urchins in all experiment diet groups increased (Table 4), especially in the sea urchins fed diets supplemented with different sources and levels of astaxanthin, suggesting that astaxanthin might be of benefit to the development of the gonads. In the present study, although no overall color assessment was carried out, no obvious difference in the gonad color of the sea urchins fed the experimental diets was observed, and the gonad color was roughly evaluated as being acceptable (a bright orange appearance) according to the method of Symonds *et al*. [35]. The evaluations were merely observational under artificial laboratory light sources, and therefore further color assessment was required by assessing colors under controlled light sources in a quantitative manner. The gonad color of wild sea urchins was highly variable and could range from a very pale yellow to a dark brown/red, in which both very pale and dark gonads were unacceptable in terms of color for the marketplace. It had been suggested that the levels of total carotenoid or individual carotenoids showed no obvious correlation with superior, acceptable, and unacceptable coloration in the gonads of sea urchins, and other, as yet unidentifiable, factors might influence the visual appearance of the gonads [35]. 

However, in terms of the diets HP1, HP2, CZ1, and CZ2, the microalgae *H. pluvialis* (2.5 and 0.6% of total diet) and *C. zofingiensis* (30.3 and 7.6% of total diet) at different percentages were added, and adding whole algae might change the nutritional composition of the diets, such as proteins, lipids, and carbohydrates, in addition to carotenoids, especially CZ1, the effect of which on the gonads could not be attributed to the level of astaxanthin alone. In addition, the diameters and weights of these sea urchins shown in Table 4 were relatively low across all treatments in comparison with the previous study on wild sea urchins *A. crassispina* [3], suggesting that the nutritional content of the diets might be insufficient for the experimental urchin utilized in this study, and a more natural diet with other key ingredients (other than just carotenoids), such as docosahexaenoic acid, might also be important.

## 5. Conclusions

The present study investigated the effects of the microalgae *H. pluvialis* containing a large amount of astaxanthin esters, *Chorella zofingiensis* containing an amount of astaxanthin esters and a small amount of canthaxanthin, and synthetic astaxanthin in the gonad of the sea urchin *A. crassispina*. The results showed that the highest astaxanthin level was detected in the sea urchins fed the diet with 380 mg/kg of added astaxanthins from *H. pluvialis*, and the highest canthaxanthin level and the lower astaxanthin level were found in the sea urchins fed the diet with 387 mg/kg of added astaxanthins and 142 mg/kg of canthaxanthin from *C. zofingiensis*. The study showed that it was possible that astaxanthin esters could not be easily assimilated by the sea urchins, and synthetic astaxanthin might not be ingested as easily as that from natural sources, so a further study should investigate the assimilation and deposit in gonads of sea urchins fed free astaxanthin from natural sources, and the carotenoid compositions in the guts and feces of sea urchins fed astaxanthin esters.
